# Secondary Structures of MERS-CoV, SARS-CoV, and SARS-CoV-2 Spike Proteins Revealed by Infrared Vibrational Spectroscopy

**DOI:** 10.3390/ijms24119550

**Published:** 2023-05-31

**Authors:** Annalisa D’Arco, Marta Di Fabrizio, Tiziana Mancini, Rosanna Mosetti, Salvatore Macis, Giovanna Tranfo, Giancarlo Della Ventura, Augusto Marcelli, Massimo Petrarca, Stefano Lupi

**Affiliations:** 1 Laboratori Nazionali Frascati, National Institute for Nuclear Physics (INFN-LNF), Via E. Fermi 54, 00044 Frascati, Italy; 2 Department of Physics, University of Rome ‘La Sapienza’, P.le A. Moro 2, 00185 Rome, Italy; 3 Laboratory of Biological Electron Microscopy, School of Basic Sciences, Institute of Physics, EPFL & Department of Fundamental Microbiology, Faculty of Biology and Medicine, UNIL, 1015 Lausanne, Switzerland; 4 Department of Occupational and Environmental Medicine, Epidemiology and Hygiene, INAIL, Monte Porzio Catone, 00078 Rome, Italy; 5 Department of Science, University Rome Tre, Largo San Leonardo Murialdo 1, 00146 Rome, Italy; 6 Rome International Centre for Materials Science Superstipes, Via dei Sabelli 119A, 00185 Rome, Italy; 7 National Institute for Nuclear Physics Section Rome1, P.le A. Moro 2, 00185 Rome, Italy; 8 Department of Basic and Applied Sciences for Engineering (SBAI), University of Rome ‘La Sapienza’, Via Scarpa 16, 00161 Rome, Italy

**Keywords:** ATR-IR spectroscopy, spike glycoproteins, secondary structure, conformation, SARS-CoV, MERS-CoV, SARS-CoV-2

## Abstract

All coronaviruses are characterized by spike glycoproteins whose S1 subunits contain the receptor binding domain (RBD). The RBD anchors the virus to the host cellular membrane to regulate the virus transmissibility and infectious process. Although the protein/receptor interaction mainly depends on the spike’s conformation, particularly on its S1 unit, their secondary structures are poorly known. In this paper, the S1 conformation was investigated for MERS-CoV, SARS-CoV, and SARS-CoV-2 at serological pH by measuring their Amide I infrared absorption bands. The SARS-CoV-2 S1 secondary structure revealed a strong difference compared to those of MERS-CoV and SARS-CoV, with a significant presence of extended β-sheets. Furthermore, the conformation of the SARS-CoV-2 S1 showed a significant change by moving from serological pH to mild acidic and alkaline pH conditions. Both results suggest the capability of infrared spectroscopy to follow the secondary structure adaptation of the SARS-CoV-2 S1 to different environments.

## 1. Introduction

The global emergency, due to the COVID-19 pandemic, poses a grave threat to public health, security, and the economy by imposing a severe burden on our society [1,2]. The virus responsible for the COVID-19 disease is a new member of the Coronaviridae family and is known as SARS-CoV-2 [2,3,4]. Like SARS-CoV-2, the other two coronaviruses (CoVs) are known to cause deadly pneumonia. These are the severe acute respiratory syndrome coronavirus (SARS-CoV) [5,6] and the Middle East respiratory syndrome coronavirus (MERS-CoV) [7], which determined the previous pandemics that occurred in 2002 and 2012, respectively [8,9]. 

These viruses have different transmissibility rates, hospitalization rates, and case fatality rates. SARS-CoV-2 is less deadly than SARS-CoV and, compared particularly to MERS-CoV, it is considered the third highly infective CoV [2,3]. However, a nodal point of any pandemic is the viral transmissibility, which is quantified by the reproductive rate parameter R_0_; if R_0_ is greater than one, the pandemic is assumed to be in a growing phase. For SARS-CoV-2, in the early months of the pandemic, the R_0_ was estimated to be between two and five [1,10] when the containment measures were not yet introduced [11,12,13]. This value was higher than those of SARS-CoV (2–3) [14,15] and MERS-CoV (0.9) [10]. 

**Figure 1:**
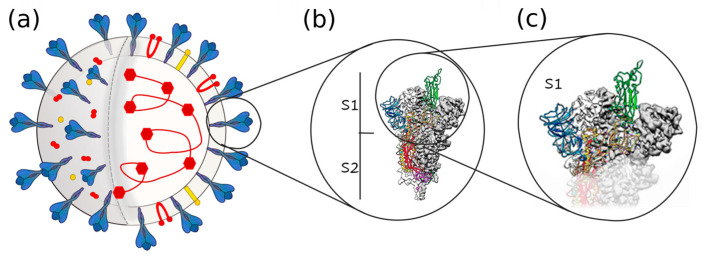
The SARS CoV-2 virion structure. (**a**) Model of the SARS-CoV-2 virion and schematic diagram: its structural proteins (spike glycoprotein S in blue, membrane protein M in red, and envelope glycoprotein E in yellow) is shown on the surface, and the nucleocapsid protein plus mRNA are shown inside the virion. (**b**) Detail of the S glycoprotein and its subunits S1 and S2. (**c**) Further detail of the S1 subunit, which was the object of this experimental study.

CoVs are spherical viruses with diameters ranging between 60–150 nm [16,17] that contain a non-segmented positive-sense RNA (+ssRNA). The RNA strain acts as an mRNA for translation of the replicase polyproteins, wherein it encodes the genetic content accessory and structural proteins, such as spike (S) glycoproteins. The spike is an I-th class fusion protein [18] protruding from the surface of mature virions (see Figure 1a). It plays a crucial role in both the infection process [19] and in viral pathogenesis [20,21], including the virus released to host cells. 

Mature S glycoproteins weigh about 150 kDa and are synthetized in terms of 1300 amino acids polypeptide chains, which associate as heavily glycosylated trimers (see Figure 1b). Each S protein is composed of two subunits (S1 and S2). The surface S1 subunit contains an N-terminal domain (NTD) and a receptor binding domain (RBD). Here, the receptor binding motif (RBM) part is responsible for anchoring the receptors [21,22], thereby triggering the endocytosis of the complex into the host cell. Once the virus receptor complex enters the cell, it is exposed to a low endosomal pH. Another structural feature of S proteins is their extensive glycosylation [23,24,25,26]. The CoV S glycoproteins are densely decorated by heterogeneous N-linked glycans that are used by viral fusion proteins as a shield to counteract the host immune response [23,27,28,29,30,31,32,33], thus participating in the S folding [34,35,36] and working as recognition sites [37]. 

The transmissibility and pathogenicity of viruses are determined by both viral and host factors, such as the combination of immune evasion, the conformational masking of binding domains, and glycan shielding, as well as the extent of the receptor binding affinity and specificity. As all CoVs present S glycoproteins, their different peculiarities, such as transmissibility, shape, and binding contacts, could be related to different secondary S protein structures [2,3,20] and to amino acid sequence variations [38]. Furthermore, another parameter that influences the secondary structure and the efficient viral propagation within a biological host is the pH [39]. It drastically changes during endocytosis: endosomal vesicles undergo rapid acidification, by transporting the virion bound to the cell membrane into the cytosol, at pHs around mildly acidic values. 

In this manuscript, we performed, for the first time, to the best of our knowledge, a comparative infrared (IR) spectroscopic study of the S1 glycoprotein monomers of MERS-CoV, SARS-CoV, and SARS-CoV-2, with the aim to investigate their complex secondary structures at a serological pH (7.4). In particular, the secondary structures of MERS-CoV, SARS-CoV, and SARS-CoV-2 S1 units were determined through a spectral component analysis of the protein amide I vibrational band [40,41,42,43,44]. Through these measurements, we experimentally demonstrated that the three S1 glycoproteins have different secondary structures. Notably, the SARS-CoV-2 S1 glycoprotein revealed a higher amount of extended β-sheet structures. This conformational difference may concern the RBM region and be indirectly correlated to the variation in the binding affinities of SARS-CoV and SARS-CoV-2 [45,46,47]. We also observed strong changes in the secondary structure of the SARS-CoV-2 S1 unit by moving from a neutral pH, which characterizes the human serological environment, to endosomal pHs (mildly acidic) and to alkaline environments. These results indicate the capability of the SARS-CoV-2 S1 glycoprotein to rapidly adapt its secondary structure to different pH environments. Our study finally demonstrates that IR vibrational spectroscopy is a valuable tool for the investigation of spike secondary conformational structure and for identifying different coronavirus families. In fact, this study represents a preliminary step towards the investigation of the spectral fingerprints of the whole virion, where the spectral complexity could be analyzed through an artificial intelligence approach [48,49].

## 2. Results

### 2.1. Amide I: Protein Secondary Structure

**Figure 2:**
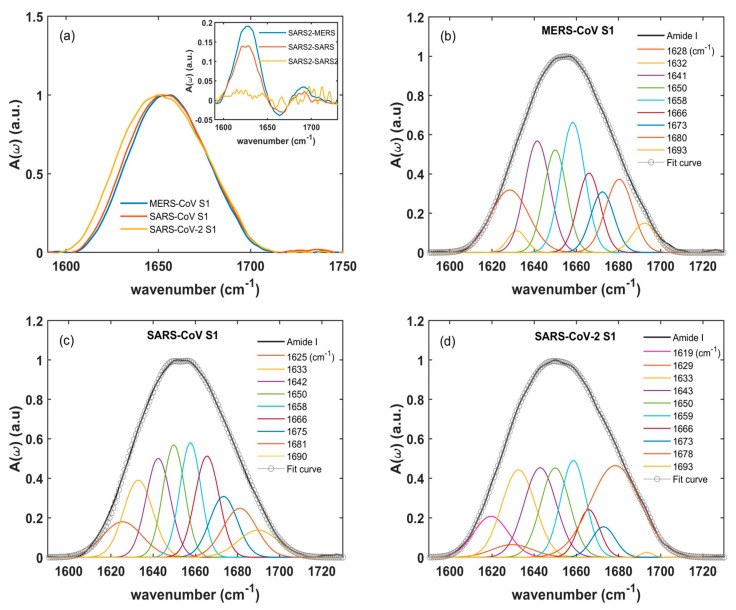
Comparison of amide I S1 absorption spectra. (**a**) A direct comparison among the S1 absorption of MERS-CoV (blue line), SARS-CoV (red line), and SARS-CoV-2 (yellow line). In the inset of the same panel, we report the differences for A(ω)_(SARS-CoV-2)_-A(ω)_(SARS-CoV)_ (red line) and A(ω)_(SARS-CoV-2)_-A(ω)_(MERS-CoV)_ (blue line) in comparison to the reproducibility of the SARS-CoV-2 absorption spectra. This was estimated by the difference for A(ω)_(SARS-CoV-2)_-A(ω)_(SARS-CoV-2)_ for two separate measurement runs (yellow line). Panels (**b**–**d**) compare the absorption spectra of MERS-CoV, SARS-CoV, and SARS-CoV-2 (black lines), as well as their decomposition based on Gaussian peaks (colored lines) and the global fitting (empty circles).

Figure 2 shows the absorbances A(ω) vs. the frequency (ω) of the S1 glycoprotein of MERS-CoV (b), SARS-CoV (c), and SARS-CoV-2 (d) of the amide I band, ranging between 1580 and 1750 cm^−1^, measured at a pH of 7.4 and at a concentration of 0.25 mg/mL. Similar data were obtained for other concentrations reported in supporting information SI (see Figure S3), where we illustrated and also discussed the absorption data of the glycan band (900–1180 cm^−1^) for a complete spectral characterization of the S1 proteins (see Figure S4 and the assignments in Table S2). For what concerns the amide I vibration, a qualitative comparison can be made by looking at Figure 2a. The MERS-CoV (blue line) and SARS-CoV (red line) showed a quite similar broad absorption band centered at about 1660 cm^−1^, while the band of SARS-CoV-2 (yellow line) had a maximum around 1650 cm^−1^. This red shift could be further quantified by calculating the differences for A(ω)_(SARS-CoV-2)_-A(ω)_(MERS-CoV)_, and A(ω)_(SARS-CoV-2)_-A(ω)_(SARS-CoV)_ (blue and red line in the inset of Figure 2a, respectively) and comparing them with the reproducibility of the A(ω)_(SARS-CoV-2)_ absorption measurements. The reproducibility was estimated by the difference for A(ω)_(SARS-CoV-2)_-A(ω)_(SARS-CoV-2)_ for two different measurement runs (yellow line in the inset of Figure 2a), with fluctuations around 2% in the whole amide I spectral range. On the other hand, a sizeable difference (actually far outside the reproducibility of the absorption spectra), was observed when comparing the SARS-CoV-2 absorption with the MERS-CoV and SARS-CoV ones. In particular, both MERS-CoV and SARS-CoV had a lower absorption intensity between 1600–1650 cm^−1^ (in agreement with the main panel of Figure 2a), and a slightly more intense signal at a higher frequency. 

In order to identify the secondary structures for MERS-CoV, SARS-CoV, and SARS-CoV-2, a global fitting approach [40,41,42,50] was used for deconvoluting the amide I band into Gaussian spectral components (as described in the section Materials and Methods). The total fit curve (empty circles) and the spectral decomposition (colored Gaussians) of their amide I bands (black lines), are reported in Figure 2b, c and d, respectively. 

Table 1 summarizes the vibrational frequencies of the different Gaussian components, their relative integrated intensities, and the assignments to specific secondary structures [40,44,50,51,52]. In particular, we noticed an intense peak around 1658 cm^−1^ associated with the α-helix structure [40,50,51,52]. The β-sheet components were observed between 1620–1640 cm^−1^ and around 1690 cm^−1^ [52]. These bands at 1630 cm^−1^ and 1690 cm^−1^ are typically related to an antiparallel arrangement of the β-sheet [40,52]. The bands located in the 1665–1680 cm^−1^ range were assigned to the β-turn structure. The broad absorption band centered at 1643 cm^−1^ corresponded to random coils. Notably, the absorption band at 1619 cm^−1^ was only present in the SARS-CoV-2 S1 unit, and it might be assigned to the extended β-sheets [40,44,50,51,52]. 

The area of each absorption band of the IR spectrum was assumed to be proportional to the relative amount of the secondary structure. Therefore, each percentage could be estimated through the ratio among the integrated intensity of its component of the amide I band over the total one [44,53] (also reported in Table 1). From these data, one can observe that SARS-CoV and SARS-CoV-2 showed similar α-helix (14.9% and 15.9%, respectively) and random coil (26.4% and 25.9%, respectively) contents. The larger difference in the secondary structures of the S1 proteins could be observed in the arrangement of the β-sheet and β-turn. A significant increase was revealed in the β-sheet contents passing from MERS-CoV (20.6%) to SARS-CoV (26.8%) and SARS-CoV-2 (30.6%). This was mainly due to the appearance of the β-sheet absorption band observed at 1619 cm^−1^ for the SARS-CoV-2 S1 unit, which corresponded to nearly 5% of the total protein secondary structure. An opposite trend was shown by the β-turn (1665–1687 cm^−1^) component. The MERS-CoV and SARS-CoV S1 units exhibited approximatively the same β-turn content (~31% and 32%, respectively) compared to ~28% for the SARS-CoV-2 S1. Although SARS-CoV-2 and SARS-CoV S1 units interact with the same receptor ACE2-peptidase and show a high value of amino acid sequence similarity (~78%, see SI) [3,54], they exhibited a robust secondary structure difference on the basis of their vibrational spectra. As the receptor protein recognition depends on the protein secondary structure, the observed differences between the SARS-CoV and SARS-CoV-2 S1 units could be related to their differential receptor-binding affinities, as several recent findings have discovered [19,55,56,57,58]. 

### 2.2. Amide I: Changes in Secondary Structure Induced by pH Variation

**Figure 3:**
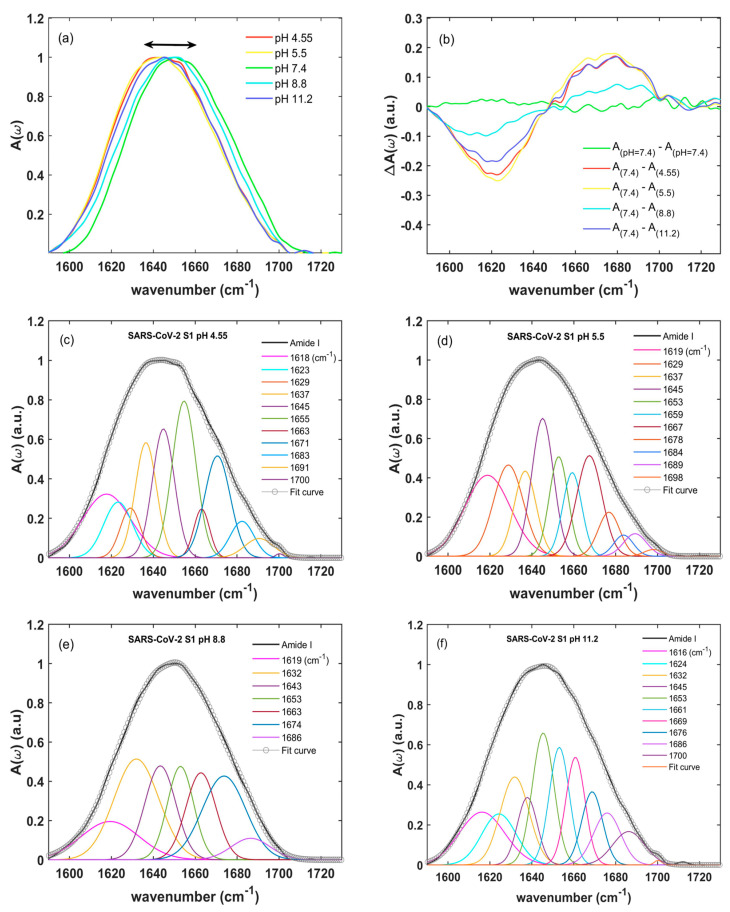
Amide I SARS-CoV-2 S1 absorption spectra at different pH values. In (**a**), a direct comparison among SARS-CoV-2 S1 absorption spectra when varying the pH from acid to alkaline conditions is shown. In (**b**), the differences for A(ω)_(pH=7.4)_-A(ω)_(pH=4.55)_ (green line), A(ω)_(pH=7.4)_-A(ω)_(pH=5.5)_ (light blue line), A(ω)_(pH=7.4)_-A(ω)_(pH=8.8)_ (violet line), and A(ω)_(pH=7.4)_-A(ω)_(pH=11.3)_ (blue line) in comparison to the reproducibility at pH = 7.4 are shown. Panels (**c**–**f**) instead show the deconvoluted absorption spectra of the SARS-CoV-2 S1 units at different pH levels (black lines) superimposed to the global fitting (empty circles) and the Gaussian component decompositions (colored lines).

In this manuscript, we also studied the variation in the secondary structure of the SARS-CoV-2 S1 unit at different pH levels (pH = 4.55, 5.5, 7.4, 8.8, and 11.2) by measuring the absorbance A(ω) of the amide I band (Figure 3a). The absorption behavior vs. pH was not monotonic. While the SARS-CoV-2 S1 spectra at mild acidic pHs (blue line at 4.55 pH and red line at 5.5 pH) were similar to the spectrum at 11.2 pH (green line), exhibiting a maximum around 1640 cm^−1^, those at the serological pH (yellow line) and pH 8.8 presented an overall blue-shifting with a maximum at about 1650 cm^−1^. These differences are highlighted in Figure 3b, where the differences for A(ω)_(pH 7.4)_-A(ω)_(pH x)_ are compared with the reproducibility of A(ω)_(pH 7.4)_. This was estimated as A(ω)_(pH=7.4)_-A(ω)_(pH=7.4)_ for two measurement runs (yellow line in Figure 3b) that showed a fluctuation of about 2% in the whole amide I spectral range. We noticed that a similar reproducibility could be observed at any pH. By varying the pH, an absorption frequency redistribution was observed around an isosbestic point at about 1647 ± 1 cm^−1^ (see Figure 3a,b). Given that the S1 protein concentrations and all physical parameters, e.g., temperature, pressure, and relative humidity, were kept constant during experiments, the occurrence of the isosbestic point could be then associated to the conformational changes in the protein structure induced by the pH. 

The influence of pH on the S1 protein conformation was quantified by studying the frequency position and the area of each spectral component of the amide I obtained from a global Gaussian fitting (reported in the SI, paragraph S4). The fit curve (empty circles) and the spectral decomposition (colored Gaussians) for different pHs (4.55, 5.5, 8.8, and 11.2) are compared in Figure 3c, d, e and f, respectively. The absorption at 7.4 pH has already been reported in Figure 2d. In the following, we will discuss the main variations in frequency and intensity of the secondary components. All peak frequencies vs. pH for the amide I S1 units, their secondary structure percentages, and their assignments are shown in Table S1. 

**Figure 4:**
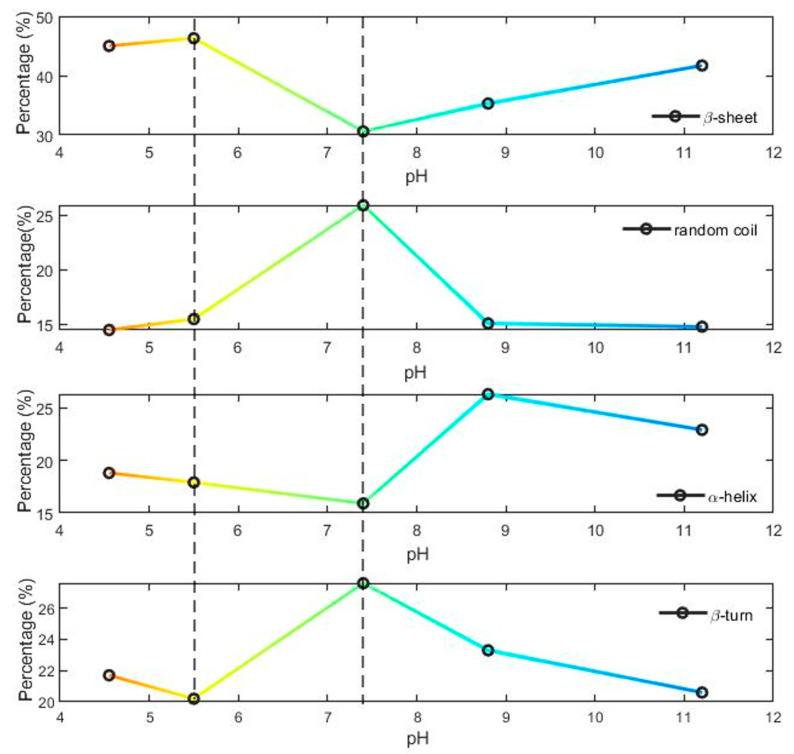
The percentages of β-sheet, α-helix, random-coil, and β-turn secondary structures vs. pHs from acid (red and yellow regions) to alkaline (cyan and blue regions) levels.

We observed significant changes in the secondary structure percentages vs. the pHs (see Figure 4) [40,44,50,52,59]. Here, each percentage was estimated through the ratio among the integrated intensity of its component of the amide I band over the total one [44,53], as described below (see Table S1 of SI). At a 7.4 pH, the percentage of α-helix (see Table S1 in SI) structures was ~16%. This value increased slightly at mild acidic pHs (~18% at 5.5 pH and ~19% at 4.55 pH), whereas a remarkable increase was observed moving to alkaline pH levels (~26%). The content of random coils was maximized at the serological pH level and instead decreased at both the acidic and alkaline levels (from ~26% at a 7.4 pH to ~15% at a 4.55 and 11.2 pH, respectively). The reduction of the random coil percentage outside the serological condition indicates a tendency to seek the minimal unfolding of the secondary structure. Moreover, the β-turn structures were affected by the pH: moving from a 7.4 pH to acidic and alkaline levels, the content of β-turn structures decreased, e.g., passing from ~28% to the minimum value of ~20% at a 5.5 pH. Conversely, we observed a slight increase in β-sheet structures at alkaline pH values, and a significant one in the mild acidic environment, with a maximum of around 46% at a 5.5 pH. In the percentage estimation of β-sheet structures, we included the contribution of the extended β-sheets (centered around 1619 cm^−1^). Its content drastically increased at a 5.5 pH with a percentage around 19% (see Table S1 in SI) with respect to the serological value (5%).

## 3. Discussion

In this paper, we investigated the secondary conformational structure of the spike glycoprotein S1, which is responsible for anchoring coronaviruses to the cellular membrane. The secondary structures of MERS-CoV, SARS-CoV, and SARS-CoV-2 S1 were characterized through IR spectroscopy by measuring their amide I vibrational bands at the serological pH. Our experiment points out that the three proteins exhibited different secondary structures. In particular, the SARS-CoV-2 S1 unit showed a significant amount of β-sheet components compared to the other spikes, with the appearance of an extended β-sheet mode at 1619 cm^−1^ that indicated a more stable protein structure [40,43,44,52]. The MERS-CoV, SARS-CoV and SARS-CoV-2 S1 units show different amino acid sequences, as determined through the Pairwise Sequence Alignment Emboss Needle (https://www.ebi.ac.uk/Tools/psa/emboss_needle/ accessed on 11 January 2023). Since a different sequence may cause a variation in protein 3D conformational structures, and, thus, in the binding affinity [38,56], in the following, we correlated our IR conformational data to the protein alignments, reported in Supporting Information (see Files S1–S6). Thus, referring to SI, we found a low sequence similarity between MERS-CoV S1 and SARS-CoV S1 units (36.4%) and between MERS-CoV S1 and SARS-CoV-2 S1 units (33.3%). The SARS-CoV and SARS-CoV-2 S1 units instead presented a level of similarity around ~78% (see SI), despite the robust secondary structure variation revealed by our vibrational absorption measurements (see above). In order to further investigate this point by comparing the NTD domains of the SARS-CoV and SARS-CoV-2 S1 units (see SI), we found a level of identity at ~73%. Instead, the identity fell to about 54% for the RBM domains. 

These analyses indicate that the structural differences between the SARS-CoV and SARS-CoV-2 S1 units observed in the infrared data should be located mainly in the protein/receptor domain. Given that the protein/receptor recognition strongly depends on the conformation, this suggests that the secondary structural differences (the presence of extended β-sheets in SARS-CoV-2 compared to SARS-CoV) are finally related to their different receptor-binding affinities and transmissibility. This fundamental result, obtained from IR data and protein alignment comparison, is in agreement with the findings of Wrapp et al. [58] and Tai et al. [19], who employed surface plasmon resonance (SPR), the ELISA test, and fluorescence, wherein they reported that the SARS-CoV-2 RBD had a higher binding affinity for the ACE2-peptidase domain than the SARS-CoV RBD [19,55,56,57,58,60,61,62,63]. 

In this manuscript, we discussed the amide I vibrational absorption of SARS-CoV-2 vs. pH. Indeed, the pH-dependent conformation changes in proteins play a key role in virus replication, pathogenesis, and transmissibility. In particular, the local environment pH strongly influences the protonation state in folded proteins by promoting changes in inter-chain interactions. Consequently, the stability of proteins is pH-dependent, thereby favoring conformational flexibility, as well as protein activation and/or inactivation. 

Our IR results (see Section 2.2) indicated a progressive rearrangement of the SARS-CoV-2 S1 unit, as a function of pH variations, when moving from the serological pH to mild acidic and alkaline values. In particular, we observed a non-monotonic behavior of the absorbance vs. pH, with a shift (~10 cm^−1^) of the broad absorption band located at 1650 cm^−1^ (due to α-helix structure) from acidic to alkaline pH values (see Figure 3a). In addition, other significant differences emerged from the spectral decomposition analysis (see above and SI). Notably, strong changes in the SARS-CoV-2 S1 secondary structure have been observed at a pH of 5.5. This is the characteristic pH value of endosomal vesicles, which facilitates successful virion entry into the cytosol. Some investigations [64,65] have shown that several regions of the SARS-CoV-2 protein are susceptible to structural modifications, with the RBD site being particularly vulnerable to a conformational change; see above and Refs. [64,65]. Indeed, the RBD undergoes a hinge-like movement that brings up or down the specific amino acid sequence responsible for the binding to the ACE2 receptor [58], inducing a change in the secondary structure. In particular, our work indicates a large variation in β-sheet content for the SARS-CoV-2 S1 vs. pH (see Figure 4), with a maximum value at a 5.5 pH of around 46%. Recent Cryo-EM microscopy works on SARS-CoV-2 glycoproteins [39,66,67] showed that the S1 protein undergoes a structural transition from a closed to a locked form as the pH is increased from mild acidic to neutral. These observations suggest a strong modification of the RBD sites vs. pHs. Combined with our IR data, this reinforces the idea that a 5.5 pH is a turning point for protein changes to its overall conformation (both its secondary and tertiary structure). The S1 reaches an optimal condition in a pre-fusion configuration at endosomal pH. Results in Refs. [39,66,67] can be therefore correlated to our IR results that reveal an increase of extended β-sheet content at pH 5.5. 

In conclusion, the secondary structures of MERS-CoV, SARS-CoV, and SARS-CoV-2 S1 units show strong differences in their amide I infrared absorption bands at the serological pH. The SARS-CoV-2 S1 secondary structure revealed the presence of extended β-sheet content in comparison to MERS-CoV and SARS-CoV, which suggests a more stable protein structure. Moreover, the conformation of the SARS-CoV-2 S1 unit showed a significant change by moving from the serological pH to mild acidic and alkaline pH conditions. Both results suggest the huge capability of IR spectroscopy to provide rapid and insightful information on the secondary structures of whole coronavirus families, thereby shedding light on their similarities and differences. Our data finally indicate the ability of the SARS-CoV-2 S1 glycoprotein to adapt to a variable environment, thus pointing out the strong role of the S1 protein secondary structure in the virus transmissibility. 

## 4. Materials and Methods

### 4.1. Protein Preparation

Recombinant S1 proteins monomers, fused with a polyhistidine tag at the C terminus of MERS-CoV (Cat. 40069-V08B1, aa 725, purity > 90%), SARS-CoV (Cat. 40150-V08B1, aa 665, purity > 90%), and SARS-CoV-2 (Cat. 40591-V08B1, aa 681, purity > 90%) were purchased from Sino Biological Europe GmbH (Eschborn, Germany). They were expressed in baculovirus insect cells with the same purity > 90% as determined by sodium dodecyl sulphate–polyacrylamide gel electrophoresis (SDS-PAGE) and finally used without further purification. The amino acid sequences and the RBM region positions are reported in SI (see Figure S1), together with the three-dimensional visualization of SARS-CoV-2 S1 protein (see Figure S2). This work was carried out on a dataset of S proteins collected in late spring 2020, and, as regards SARS-CoV-2, we referred to the alpha variant that affected Europe and Italy in the pandemic crisis of March 2020. The lyophilized proteins were reconstructed by dissolving 100 µg in distilled water (400 µL) at pH 7.4 (0.25 mg/mL concentration). The samples for the pH-dependent study of SARS-CoV-2 S1 subunit were prepared as follows. Briefly, in order to adjust the pH of proteins, NaOH or HCl aqueous solutions at molar concentrations of 10^−2^ and 10^−4^ M were used. The solutions were gently shaken while waiting for the state of equilibrium of the reaction, and the pH was monitored with a pH meter from DOSTMANN (Wertheim, Germany) pH 80+ DHS. We prepared two samples of acidic S1 protein solutions at pH 4.55 and 5.5, and two in alkaline environments, having pH 8.8 and 11.2. 

### 4.2. Attenuated-Total-Reflection Infrared Spectroscopy and Data Analysis

Attenuated total reflection (ATR) infrared spectra of the Spike glycoproteins S1 units of MERS-CoV, SARS-CoV, and SARS-CoV-2 were collected using a Bruker (Billerica, MA, USA) Vertex 70v Michelson spectrometer equipped with an ATR–Diamond module Harrick (Pleasantville, NY, USA) MVP-Pro and a DLaTGS wide range detector. Spectroscopic measurements were carried out at room temperature and with the interferometer under vacuum in order to eliminate water vapor and CO2 absorptions. The background spectrum (buffer solution) was collected immediately prior to each sample measurement. Five microliters of the sample solutions was placed directly on the diamond crystal, and 64 scans between 400–4000 cm^−1^ with a resolution of 2 cm^−1^ were acquired. Each spectrum was the average of six independent measurements. The ATR crystal was cleaned with ethanol (added purity) and subsequently with a lens tissue in order to eliminate any spurious signal. Raw data were processed and analyzed using OPUS 8.2 (Bruker Optics) and in-house algorithms based on MATLAB (ver. 2018, MathWorks Inc., Natick, MA, USA). To obtain the protein absorption spectra A(ω), shown in Figure 2, we subtracted the buffer spectrum (see supplementary information for details) to eliminate the contribution of the background [50,51] and applied the ATR correction algorithm and a piecewise linear baseline subtraction. The secondary structures of glycoprotein S1 units were obtained by the decomposition of the amide I vibrational absorption band [41,44] into its spectral components. Several numerical treatments were adopted to decompose the amide I band, thereby facilitating the analysis of frequency–structure correlations in the vibrational spectroscopy of proteins, such as Fourier self-deconvolution, factor analysis, and excitonic theory [50,51,52,68,69]. In this case, we deconvolved the spectra through the 2nd-derivative procedure [50,52] combined with a multicomponent Gaussian fitting. In particular, the frequencies, achieved by 2nd-derivative spectra, were used as starting points for Gaussian curve fitting, and the residual error (RMSE) was employed for assessing the convolution procedure performance. The intensity of each component peak, normalized to the total intensity, was used to calculate the percentage of each absorption band and then to estimate the secondary structures of the S1 unit spike proteins [44,53,59,70]. The band assignment of glycoproteins in the amide I region was assigned according to the literature [40,50,52]: bands between 1653 cm^−1^ and 1659 cm^−1^ were referred to as α-helix; bands ranging 1640–1650 cm^−1^ were assigned to random coils; bands between 1662 cm^−1^ and 1686 cm^−1^ were assigned to β-turns, and bands from 1687 cm^−1^ to 1696 cm^−1^ and from 1617 cm^−1^ were assigned to 1638 cm^−1^ to β-sheets. 

## Figures and Tables

**Table 1:** Secondary structure assignment for MERS-CoV, SARS-CoV, and SARS-CoV-2 S1 units derived from the Gaussian decomposition of the vibrational absorption spectra [40,52].

MERS-CoV Peak Frequency [cm^−1^]	MERS-CoV Relative Integrated Intensity [%]	SARS-CoV Peak Frequency [cm^−1^]	SARS-CoV Relative Integrated Intensity [%]	SARS-CoV-2 Peak Frequency [cm^−1^]	SARS-CoV-2 Relative Integrated Intensity [%]	Assignment
-	-	-	-	1619	5.2	β-sheet (extended)
1628	14.0	1625	7.9	1628	11.4	β-sheet
1632	2.4	1633	12.7	1633	9.8	β-sheet
1641	16.5	1642	13.5	1643	12.3	Random coils
1650	13.7	1650	12.9	1650	13.6	Random coils
1658	18.6	1658	14.9	1659	15.9	α-helix
1666	10.6	1666	12.2	1666	5.6	β-turn
1673	8.3	-	-	1673	6.7	β-turn
-	-	1675	10.4	-	-	β-turn
1680	11.8	1681	9.3	1678	15.2	β-turn
1693	4.2	1690	6.1	1693	4.2	β-sheet

## Data Availability

The data presented in this study are available on request from the corresponding authors.

## Supplementary Materials

The following supporting information can be downloaded at: https://www.mdpi.com/article/10.3390/ijms24119550/s1. Refs. [71,72,73] are cited in the Supplementary Material.
