# Supplementary material for: Secondary Structures of MERS-CoV, SARS-CoV, and SARS-CoV-2 Spike Proteins Revealed by Infrared Vibrational Spectroscopy
Source: Int J Mol Sci. 2023 May 31;24(11):9550. doi: 10.3390/ijms24119550 (PMC10253540; doi:10.3390/ijms24119550)
Supplement: Supplementary file 1 [file ijms-24-09550-s001.zip › File S1.pdf]

```
########################################
# Program: needle
# Rundate: Wed 11 Jan 2023 08:39:38
# Commandline: needle
#    -auto
#    -stdout
#    -asequence emboss_needle-I20230111-083937-0108-9752-p2m.asequence
#    -bsequence emboss_needle-I20230111-083937-0108-9752-p2m.bsequence
#    -datafile EBLOSUM62
#    -gapopen 10.0
#    -gapextend 0.5
#    -endopen 10.0
#    -endextend 0.5
#    -aformat3 pair
#    -sprotein1
#    -sprotein2
# Align_format: pair
# Report_file: stdout
########################################

#=======================================
#
# Aligned_sequences: 2
# 1: MERS-CoV
# 2: SARS-CoV
# Matrix: EBLOSUM62
# Gap_penalty: 10.0
# Extend_penalty: 0.5
#
# Length: 803
# Identity:     190/803 (23.7%)
# Similarity:   292/803 (36.4%)
# Gaps:         222/803 (27.6%)
# Score: 383.5
#
#
#=======================================

MERS-CoV           1 YVDVGPDSVKSACIEVDIQQTFFDKTWPRPID-VSKADGIIYPQGRTYSN     49
                                  .::|...||.|...|.... .|...|:.||.....|:
SARS-CoV           1 -------------SDLDRCTTFDDVQAPNYTQHTSSMRGVYYPDEIFRSD     37

MERS-CoV          50 ITITYQGLF-PYQGDHGDMYVYSAGHATGTTPQKLFVANYSQD--VKQFA     96
                     .....|.|| |:         ||  :.||     ....|::.|  |..|.
SARS-CoV          38 TLYLTQDLFLPF---------YS--NVTG-----FHTINHTFDNPVIPFK     71

MERS-CoV          97 NGF---------VVR---IGAAAN--STGTVIISPSTSATIRKI------    126
                     :|.         |||   .|:..|  |...:||:.||:..||..
SARS-CoV          72 DGIYFAATEKSNVVRGWVFGSTMNNKSQSVIIINNSTNVVIRACNFELCD    121

MERS-CoV         127 YPAFMLGSSVGNFSDGKMGRFFNHTLVLLPDGCGTLLRAFYCILEPRSGN    176
                     .|.|.:...:|..:         ||::.  |      .||.|..|
SARS-CoV         122 NPFFAVSKPMGTQT---------HTMIF--D------NAFNCTFE-----    149

MERS-CoV         177 HCPAGNSYTSFATYHTPATDCSD--GNYNRNASLNSFKEYFNLRNCTFMY    224
                            |.|.|.    :.|.|:  ||:..      .:|:.......|:|
SARS-CoV         150 -------YISDAF----SLDVSEKSGNFKH------LREFVFKNKDGFLY    182

MERS-CoV         225 TY------NITEDEILEWFGITQTAQGVHLFSSRYVDLYGGNMFQFATL-    267
                     .|      ::..|          ...|.:.....:....|.|:..|..:
SARS-CoV         183 VYKGYQPIDVVRD----------LPSGFNTLKPIFKLPLGINITNFRAIL    222

MERS-CoV         268 ----PVYDTIKYYSIIPHSIRSIQSDRKAW----AAFYVYKLQPLTFLLD    309
                         |..||                    |    ||::|..|:|.||:|.
SARS-CoV         223 TAFSPAQDT--------------------WGTSAAAYFVGYLKPTTFMLK    252

MERS-CoV         310 FSVDGYIRRAIDCGFNDLSQLHCSYESFDVESGVYSVSSFEAKPSGSVVE    359
                     :..:|.|..|:||..|.|::|.||.:||:::.|:|..|:|...|||.||.
```

```
SARS-CoV         253 YDENGTITDAVDCSQNPLAELKCSVKSFEIDKGIYQTSNFRVVPSGDVVR    302

MERS-CoV         360 QAEGVE-CDFSPLLSGTP-PQVYNFKRLVFTNC--NYNLTKLLSLFSVND    405
                     ...... |.|..:.:.|. |.||.::|...:||  :|::....:.||.
SARS-CoV         303 FPNITNLCPFGEVFNATKFPSVYAWERKKISNCVADYSVLYNSTFFST--    350

MERS-CoV         406 FTCSQISPAAIASNCYSSLILDYFSYPLSMKSD----LSVSSAGPISQFN    451
                     |.|..:|...:...|:|::..|.|    .:|.|    ::....|.|:.:|
SARS-CoV         351 FKCYGVSATKLNDLCFSNVYADSF----VVKGDDVRQIAPGQTGVIADYN    396

MERS-CoV         452 YKQSFSNPTCLILATVPHNL---TTITKPLKYSYINKCS-RLLSDDRTEV    497
                     ||.......| :||....|:   :|.....||.|:.... |....|.:.|
SARS-CoV         397 YKLPDDFMGC-VLAWNTRNIDATSTGNYNYKYRYLRHGKLRPFERDISNV    445

MERS-CoV         498 PQLVNANQYSPCVSIVPSTVWEDGDYYRKQLSPLEGGGWLVASGSTVAMT    547
                     |...:.   .||.....:..|...||           |:...:|......
SARS-CoV         446 PFSPDG---KPCTPPALNCYWPLNDY-----------GFYTTTGIGYQPY    481

MERS-CoV         548 EQLQMGFGITVQYGTDTNSVC-PKLEFANDTKIASQLGNCVEYSLYGVSG    596
                     ..:.:.|.:.....|    || |||   :...|.:|   ||.::..|::|
SARS-CoV         482 RVVVLSFELLNAPAT----VCGPKL---STDLIKNQ---CVNFNFNGLTG    521

MERS-CoV         597 RGVFQNCTAVGVRQQRFVYDAYQNLVGYYSDD------GNYYCLRACVSV    640
                     .||.   |....|.|.|  ..:...|..::|.      .....:..|...
SARS-CoV         522 TGVL---TPSSKRFQPF--QQFGRDVSDFTDSVRDPKTSEILDISPCSFG    566

MERS-CoV         641 PVSVIY--DKETKTHATLFGSVACEHISSTM--SQYSRSTRSMLKRRDST    686
                     .||||.  ...:...|.|:..|.|..:|:.:  .|.:.:.| :....::.
SARS-CoV         567 GVSVITPGTNASSEVAVLYQDVNCTDVSTAIHADQLTPAWR-IYSTGNNV    615

MERS-CoV         687 YGPLQTPVGCVLGL--VNSSL------------------FVEAHHHHHHH    716
                     :   ||..||::|.  |::|.                  .:.||||||||
SARS-CoV         616 F---QTQAGCLIGAEHVDTSYECDIPIGAGICASYHTVSLLRAHHHHHHH    662

MERS-CoV         717 HHH    719
                     |||
SARS-CoV         663 HHH    665


#---------------------------------------
#---------------------------------------
```
