# Supplementary material for: Secondary Structures of MERS-CoV, SARS-CoV, and SARS-CoV-2 Spike Proteins Revealed by Infrared Vibrational Spectroscopy
Source: Int J Mol Sci. 2023 May 31;24(11):9550. doi: 10.3390/ijms24119550 (PMC10253540; doi:10.3390/ijms24119550)
Supplement: Supplementary file 1 [file ijms-24-09550-s001.zip › File S2.pdf]

```
########################################
# Program: needle
# Rundate: Wed 11 Jan 2023 08:39:44
# Commandline: needle
#    -auto
#    -stdout
#    -asequence emboss_needle-I20230111-084326-0712-42509776-p1m.asequence
#    -bsequence emboss_needle-I20230111-084326-0712-42509776-p1m.bsequence
#    -datafile EBLOSUM62
#    -gapopen 10.0
#    -gapextend 0.5
#    -endopen 10.0
#    -endextend 0.5
#    -aformat3 pair
#    -sprotein1
#    -sprotein2
# Align_format: pair
# Report_file: stdout
########################################

#=======================================
#
# Aligned_sequences: 2
# 1: SARS-CoV-2
# 2: MERS-CoV
# Matrix: EBLOSUM62
# Gap_penalty: 10.0
# Extend_penalty: 0.5
#
# Length: 835
# Identity:     169/835 (20.2%)
# Similarity:   278/835 (33.3%)
# Gaps:         270/835 (32.3%)
# Score: 373.5
#
#
#=======================================

SARS-CoV-2         1 ------------------------------VNLTTRTQLPPAYTNSFTRG     20
                                                   :::            |...|
MERS-CoV           1 YVDVGPDSVKSACIEVDIQQTFFDKTWPRPIDV------------SKADG     38

SARS-CoV-2        21 VYYPDKVFRSSVLHSTQDLFLPF---FSNVTWFHAIHVSGTNGTKRF---     64
                     :.||.....|::..:.|.|| |:   ..::..:.|.|.:||...|.|
MERS-CoV          39 IIYPQGRTYSNITITYQGLF-PYQGDHGDMYVYSAGHATGTTPQKLFVAN     87

SARS-CoV-2        65 -DNPVLPF-------------NDGVYFASTEKSNIIR--------GWIFG     92
                      ...|..|             :.|....|...|..||        |...|
MERS-CoV          88 YSQDVKQFANGFVVRIGAAANSTGTVIISPSTSATIRKIYPAFMLGSSVG    137

SARS-CoV-2        93 TTLDSK-----TQSLLIVNNATNVVIKVCEFQFCNDPFLGVY--------    129
                     ...|.|     ..:|:::.:....:::.  |....:|..|.:
MERS-CoV         138 NFSDGKMGRFFNHTLVLLPDGCGTLLRA--FYCILEPRSGNHCPAGNSYT    185

SARS-CoV-2       130 ----YH---------KNNKSWMESEFRVYSSANNCTFEY---VSQPFLMD    163
                         ||         ..|::...:.|:.|.:..||||.|   :::..:::
MERS-CoV         186 SFATYHTPATDCSDGNYNRNASLNSFKEYFNLRNCTFMYTYNITEDEILE    235

SARS-CoV-2       164 LEGKQGNFKNLREFVFKNIDGYFKIYSKHTPINLVRDLPQGFSALEPLVD    213
                     ..|.....:.:..|..:.:|.|
MERS-CoV         236 WFGITQTAQGVHLFSSRYVDLY----------------------------    257

SARS-CoV-2       214 LPIGINITRFQTL---------LALHRSYLTPGDSSSGWTAGAAAYYVGY    254
                        |.|:.:|.||         ..:..|..:.......|    ||:||..
MERS-CoV         258 ---GGNMFQFATLPVYDTIKYYSIIPHSIRSIQSDRKAW----AAFYVYK    300

SARS-CoV-2       255 LQPRTFLLKYNENGTITDAVDCALDPLSETKCTLKSFTVEKGIYQTSNFR    304
                     |||.||||.::.:|.|..|:||..:.||:..|:.:||.||.|:|..|:|.
```

```
MERS-CoV          301 LQPLTFLLDFSVDGYIRRAIDCGFNDLSQLHCSYESFDVESGVYSVSSFE    350

SARS-CoV-2        305 VQPTESIVRFPNITNLCPFGEVFNATRFASVYAWNRKRISNCVADYSVLY    354
                      .:|:.|:|....... |.|..:.:.|. ..||.:.|...:||..:.:.|.
MERS-CoV          351 AKPSGSVVEQAEGVE-CDFSPLLSGTP-PQVYNFKRLVFTNCNYNLTKLL    398

SARS-CoV-2        355 NSASFSTFKCYGVSPTKLNDLCFTNVYADSFVIRGDEVRQIAPGQTGKIA    404
                      :..|.:.|.|..:||..:...|::::..|.|.........:.....|.|:
MERS-CoV          399 SLFSVNDFTCSQISPAAIASNCYSSLILDYFSYPLSMKSDLSVSSAGPIS    448

SARS-CoV-2        405 DYNYKLPDDFTGC-VIAWNSNNLDSKVGG-NYNYLYRLFRKSNLKPFERD    452
                      .:|||.......| ::|...:||.:.... .|:|:.:..|..:      |
MERS-CoV          449 QFNYKQSFSNPTCLILATVPHNLTTITKPLKYSYINKCSRLLS------D    492

SARS-CoV-2        453 ISTEIYQAGS----TPCNGV------EGFNCY----FPLQSYGFQPTNGV    488
                      ..||:.|..:    :||..:      |..:.|    .||:..|:...:|.
MERS-CoV          493 DRTEVPQLVNANQYSPCVSIVPSTVWEDGDYYRKQLSPLEGGGWLVASGS    542

SARS-CoV-2        489 GYQPYRVVVLSFELLHAPAT----VCGPKKSTNLVK-----NKCVNFNFN    529
                      .......:.:.|.:.....|    ||...:..|..|     ..||.::..
MERS-CoV          543 TVAMTEQLQMGFGITVQYGTDTNSVCPKLEFANDTKIASQLGNCVEYSLY    592

SARS-CoV-2        530 GLTGTGVLTESNKKFLPFQQFGRDIADTTDAVRDPQTLEILDITPCSFGG    579
                      |::|.||........:..|:|      ..||.:
MERS-CoV          593 GVSGRGVFQNCTAVGVRQQRF------VYDAYQ-----------------    619

SARS-CoV-2        580 VSVITPGTNTSNQVAVLYQDVN------CTEVPVAI--------HA----    611
                                 |.|.....|.|      |..|||::        ||
MERS-CoV          620 -----------NLVGYYSDDGNYYCLRACVSVPVSVIYDKETKTHATLFG    658

SARS-CoV-2        612 ----DQLTPTWRVYSTGS-----------NVFQTRAGCLIGAEHVNNSYE    646
                          :.::.|...||..:           ...||..||::|.
MERS-CoV          659 SVACEHISSTMSQYSRSTRSMLKRRDSTYGPLQTPVGCVLGL--------    700

SARS-CoV-2        647 CDIPIGAGICASYQTQTNSPRRARAHHHHHHHHHH    681
                                      .||.....|||||||||||
MERS-CoV          701 ----------------VNSSLFVEAHHHHHHHHHH    719


#---------------------------------------
#---------------------------------------
```
