# Supplementary material for: Secondary Structures of MERS-CoV, SARS-CoV, and SARS-CoV-2 Spike Proteins Revealed by Infrared Vibrational Spectroscopy
Source: Int J Mol Sci. 2023 May 31;24(11):9550. doi: 10.3390/ijms24119550 (PMC10253540; doi:10.3390/ijms24119550)
Supplement: Supplementary file 1 [file ijms-24-09550-s001.zip › File S3.pdf]

```
########################################
# Program: needle
# Rundate: Wed 11 Jan 2023 08:48:16
# Commandline: needle
#    -auto
#    -stdout
#    -asequence emboss_needle-I20230111-084739-0314-81520469-p1m.asequence
#    -bsequence emboss_needle-I20230111-084739-0314-81520469-p1m.bsequence
#    -datafile EBLOSUM62
#    -gapopen 10.0
#    -gapextend 0.5
#    -endopen 10.0
#    -endextend 0.5
#    -aformat3 pair
#    -sprotein1
#    -sprotein2
# Align_format: pair
# Report_file: stdout
########################################

#=======================================
#
# Aligned_sequences: 2
# 1: SARS-CoV-2
# 2: SARS-CoV
# Matrix: EBLOSUM62
# Gap_penalty: 10.0
# Extend_penalty: 0.5
#
# Length: 299
# Identity:     160/299 (53.5%)
# Similarity:   219/299 (73.2%)
# Gaps:          27/299 ( 9.0%)
# Score: 881.0
#
#
#=======================================

SARS-CoV-2         1 -------TNSFTRGVYYPDKVFRSSVLHSTQDLFLPFFSNVTWFHAIHVS     43
                            |:|. |||||||::|||..|:.||||||||:||||.||.|
SARS-CoV           1 APNYTQHTSSM-RGVYYPDEIFRSDTLYLTQDLFLPFYSNVTGFHTI---     46

SARS-CoV-2        44 GTNGTKRFDNPVLPFNDGVYFASTEKSNIIRGWIFGTTLDSKTQSLLIVN     93
                       |.|  |||||:||.||:|||:|||||::|||:||:|:::|:||::|:|
SARS-CoV          47 --NHT--FDNPVIPFKDGIYFAATEKSNVVRGWVFGSTMNNKSQSVIIIN     92

SARS-CoV-2        94 NATNVVIKVCEFQFCNDPFLGVYYHKNNKSWMESEFRVYSSANNCTFEYV    143
                     |:|||||:.|.|:.|::||..|    :.....::...::.:|.||||||:
SARS-CoV          93 NSTNVVIRACNFELCDNPFFAV----SKPMGTQTHTMIFDNAFNCTFEYI    138

SARS-CoV-2       144 SQPFLMDLEGKQGNFKNLREFVFKNIDGYFKIYSKHTPINLVRDLPQGFS    193
                     |..|.:|:..|.||||:||||||||.||:..:|..:.||::|||||.||:
SARS-CoV         139 SDAFSLDVSEKSGNFKHLREFVFKNKDGFLYVYKGYQPIDVVRDLPSGFN    188

SARS-CoV-2       194 ALEPLVDLPIGINITRFQTLLALHRSYLTPGDSSSGWTAGAAAYYVGYLQ    243
                     .|:|:..||:|||||.|:.:|    :..:|...:  |...||||:||||:
SARS-CoV         189 TLKPIFKLPLGINITNFRAIL----TAFSPAQDT--WGTSAAAYFVGYLK    232

SARS-CoV-2       244 PRTFLLKYNENGTITDAVDCALDPLSETKCTLKSFTVEKGIYQTSNFRV    292
                     |.||:|||:|||||||||||:.:||:|.||::|||.::|||||||||
SARS-CoV         233 PTTFMLKYDENGTITDAVDCSQNPLAELKCSVKSFEIDKGIYQTSNF--    279


#---------------------------------------
#---------------------------------------
```
