# Supplementary material for: Secondary Structures of MERS-CoV, SARS-CoV, and SARS-CoV-2 Spike Proteins Revealed by Infrared Vibrational Spectroscopy
Source: Int J Mol Sci. 2023 May 31;24(11):9550. doi: 10.3390/ijms24119550 (PMC10253540; doi:10.3390/ijms24119550)
Supplement: Supplementary file 1 [file ijms-24-09550-s001.zip › File S4.pdf]

```
########################################
# Program: needle
# Rundate: Wed 11 Jan 2023 08:51:44
# Commandline: needle
#    -auto
#    -stdout
#    -asequence emboss_needle-I20230111-084953-0614-78224635-p1m.asequence
#    -bsequence emboss_needle-I20230111-084953-0614-78224635-p1m.bsequence
#    -datafile EBLOSUM62
#    -gapopen 10.0
#    -gapextend 0.5
#    -endopen 10.0
#    -endextend 0.5
#    -aformat3 pair
#    -sprotein1
#    -sprotein2
# Align_format: pair
# Report_file: stdout
########################################

#=======================================
#
# Aligned_sequences: 2
# 1: SARS-CoV-2
# 2: SARS-CoV
# Matrix: EBLOSUM62
# Gap_penalty: 10.0
# Extend_penalty: 0.5
#
# Length: 225
# Identity:     165/225 (73.3%)
# Similarity:   183/225 (81.3%)
# Gaps:           5/225 ( 2.2%)
# Score: 911.0
#
#
#=======================================

SARS-CoV-2         1 --NLCPFGEVFNATRFASVYAWNRKRISNCVADYSVLYNSASFSTFKCYG     48
                       ||||||||||||:|.|||||.||:||||||||||||||..||||||||
SARS-CoV           1 ITNLCPFGEVFNATKFPSVYAWERKKISNCVADYSVLYNSTFFSTFKCYG     50

SARS-CoV-2        49 VSPTKLNDLCFTNVYADSFVIRGDEVRQIAPGQTGKIADYNYKLPDDFTG     98
                     ||.||||||||:||||||||::||:||||||||||.||||||||||||.|
SARS-CoV          51 VSATKLNDLCFSNVYADSFVVKGDDVRQIAPGQTGVIADYNYKLPDDFMG    100

SARS-CoV-2        99 CVIAWNSNNLDSKVGGNYNYLYRLFRKSNLKPFERDISTEIYQAGSTPCN    148
                     ||:|||:.|:|:...|||||.||..|...|:|||||||...:.....||.
SARS-CoV         101 CVLAWNTRNIDATSTGNYNYKYRYLRHGKLRPFERDISNVPFSPDGKPCT    150

SARS-CoV-2       149 GVEGFNCYFPLQSYGFQPTNGVGYQPYRVVVLSFELLHAPATVCGPKKST    198
                      ....|||:||..|||..|.|:|||||||||||||||:|||||||||.||
SARS-CoV         151 -PPALNCYWPLNDYGFYTTTGIGYQPYRVVVLSFELLNAPATVCGPKLST    199

SARS-CoV-2       199 NLVKNKCVNFNFNGLTGTGVLTESN    223
                     :|:||:||||||||||||||||.
SARS-CoV         200 DLIKNQCVNFNFNGLTGTGVLTP--    222


#---------------------------------------
#---------------------------------------
```
