# Supplementary material for: Secondary Structures of MERS-CoV, SARS-CoV, and SARS-CoV-2 Spike Proteins Revealed by Infrared Vibrational Spectroscopy
Source: Int J Mol Sci. 2023 May 31;24(11):9550. doi: 10.3390/ijms24119550 (PMC10253540; doi:10.3390/ijms24119550)
Supplement: Supplementary file 1 [file ijms-24-09550-s001.zip › File S5.pdf]

```
########################################
# Program: needle
# Rundate: Wed 11 Jan 2023 08:51:55
# Commandline: needle
#    -auto
#    -stdout
#    -asequence emboss_needle-I20230111-085117-0674-7138694-p1m.asequence
#    -bsequence emboss_needle-I20230111-085117-0674-7138694-p1m.bsequence
#    -datafile EBLOSUM62
#    -gapopen 10.0
#    -gapextend 0.5
#    -endopen 10.0
#    -endextend 0.5
#    -aformat3 pair
#    -sprotein1
#    -sprotein2
# Align_format: pair
# Report_file: stdout
########################################

#=======================================
#
# Aligned_sequences: 2
# 1: SARS-CoV-2
# 2: SARS-CoV
# Matrix: EBLOSUM62
# Gap_penalty: 10.0
# Extend_penalty: 0.5
#
# Length: 74
# Identity:      40/74 (54.1%)
# Similarity:    45/74 (60.8%)
# Gaps:           5/74 ( 6.8%)
# Score: 207.0
#
#
#=======================================

SARS-CoV-2         1 --LYRLFRKSNLKPFERDISTEIYQAGSTPCNGVEGFNCYFPLQSYGFQP     48
                       .||..|...|:|||||||...:.....||. ....|||:||..|||..
SARS-CoV           1 NYKYRYLRHGKLRPFERDISNVPFSPDGKPCT-PPALNCYWPLNDYGFYT     49

SARS-CoV-2        49 TNGVGYQPYRVVVLSFELLHAPAT     72
                     |.|:|||||||||||||||:||
SARS-CoV          50 TTGIGYQPYRVVVLSFELLNAP--     71


#---------------------------------------
#---------------------------------------
```
