# Supplementary material for: Secondary Structures of MERS-CoV, SARS-CoV, and SARS-CoV-2 Spike Proteins Revealed by Infrared Vibrational Spectroscopy
Source: Int J Mol Sci. 2023 May 31;24(11):9550. doi: 10.3390/ijms24119550 (PMC10253540; doi:10.3390/ijms24119550)
Supplement: Supplementary file 1 [file ijms-24-09550-s001.zip › File S6.pdf]

```
########################################
# Program: needle
# Rundate: Wed 11 Jan 2023 08:38:40
# Commandline: needle
#    -auto
#    -stdout
#    -asequence emboss_needle-I20230111-084136-0084-12866431-p1m.asequence
#    -bsequence emboss_needle-I20230111-084136-0084-12866431-p1m.bsequence
#    -datafile EBLOSUM62
#    -gapopen 10.0
#    -gapextend 0.5
#    -endopen 10.0
#    -endextend 0.5
#    -aformat3 pair
#    -sprotein1
#    -sprotein2
# Align_format: pair
# Report_file: stdout
########################################

#=======================================
#
# Aligned_sequences: 2
# 1: SARS-CoV-2
# 2: SARS-CoV
# Matrix: EBLOSUM62
# Gap_penalty: 10.0
# Extend_penalty: 0.5
#
# Length: 692
# Identity:     449/692 (64.9%)
# Similarity:   544/692 (78.6%)
# Gaps:          38/692 ( 5.5%)
# Score: 2470.0
#
#
#=======================================

SARS-CoV-2         1 -----------VNLTTRTQLPPAYTNSFTRGVYYPDKVFRSSVLHSTQDL     39
                                |.....||    :|:|. |||||||::|||..|:.||||
SARS-CoV           1 SDLDRCTTFDDVQAPNYTQ----HTSSM-RGVYYPDEIFRSDTLYLTQDL     45

SARS-CoV-2        40 FLPFFSNVTWFHAIHVSGTNGTKRFDNPVLPFNDGVYFASTEKSNIIRGW     89
                     ||||:||||.||.|     |.|  |||||:||.||:|||:|||||::|||
SARS-CoV          46 FLPFYSNVTGFHTI-----NHT--FDNPVIPFKDGIYFAATEKSNVVRGW     88

SARS-CoV-2        90 IFGTTLDSKTQSLLIVNNATNVVIKVCEFQFCNDPFLGVYYHKNNKSWME    139
                     :||:|:::|:||::|:||:|||||:.|.|:.|::||..|    :.....:
SARS-CoV          89 VFGSTMNNKSQSVIIINNSTNVVIRACNFELCDNPFFAV----SKPMGTQ    134

SARS-CoV-2       140 SEFRVYSSANNCTFEYVSQPFLMDLEGKQGNFKNLREFVFKNIDGYFKIY    189
                     :...::.:|.||||||:|..|.:|:..|.||||:||||||||.||:..:|
SARS-CoV         135 THTMIFDNAFNCTFEYISDAFSLDVSEKSGNFKHLREFVFKNKDGFLYVY    184

SARS-CoV-2       190 SKHTPINLVRDLPQGFSALEPLVDLPIGINITRFQTLLALHRSYLTPGDS    239
                     ..:.||::|||||.||:.|:|:..||:|||||.|:.:|    :..:|...
SARS-CoV         185 KGYQPIDVVRDLPSGFNTLKPIFKLPLGINITNFRAIL----TAFSPAQD    230

SARS-CoV-2       240 SSGWTAGAAAYYVGYLQPRTFLLKYNENGTITDAVDCALDPLSETKCTLK    289
                     :  |...||||:||||:|.||:|||:|||||||||||:.:||:|.||::|
SARS-CoV         231 T--WGTSAAAYFVGYLKPTTFMLKYDENGTITDAVDCSQNPLAELKCSVK    278

SARS-CoV-2       290 SFTVEKGIYQTSNFRVQPTESIVRFPNITNLCPFGEVFNATRFASVYAWN    339
                     ||.::|||||||||||.|:..:|||||||||||||||||||:|.|||||.
SARS-CoV         279 SFEIDKGIYQTSNFRVVPSGDVVRFPNITNLCPFGEVFNATKFPSVYAWE    328

SARS-CoV-2       340 RKRISNCVADYSVLYNSASFSTFKCYGVSPTKLNDLCFTNVYADSFVIRG    389
                     ||:||||||||||||||..||||||||||.||||||||:||||||||::|
```

```
SARS-CoV         329 RKKISNCVADYSVLYNSTFFSTFKCYGVSATKLNDLCFSNVYADSFVVKG    378

SARS-CoV-2       390 DEVRQIAPGQTGKIADYNYKLPDDFTGCVIAWNSNNLDSKVGGNYNYLYR    439
                     |:||||||||||.||||||||||||.|||:|||:.|:|:...|||||.||
SARS-CoV         379 DDVRQIAPGQTGVIADYNYKLPDDFMGCVLAWNTRNIDATSTGNYNYKYR    428

SARS-CoV-2       440 LFRKSNLKPFERDISTEIYQAGSTPCNGVEGFNCYFPLQSYGFQPTNGVG    489
                     ..|...|:|||||||...:.....||. ....|||:||..|||..|.|:|
SARS-CoV         429 YLRHGKLRPFERDISNVPFSPDGKPCT-PPALNCYWPLNDYGFYTTTGIG    477

SARS-CoV-2       490 YQPYRVVVLSFELLHAPATVCGPKKSTNLVKNKCVNFNFNGLTGTGVLTE    539
                     ||||||||||||||:|||||||||.||:|:||:||||||||||||||||.
SARS-CoV         478 YQPYRVVVLSFELLNAPATVCGPKLSTDLIKNQCVNFNFNGLTGTGVLTP    527

SARS-CoV-2       540 SNKKFLPFQQFGRDIADTTDAVRDPQTLEILDITPCSFGGVSVITPGTNT    589
                     |:|:|.||||||||::|.||:||||:|.|||||:|||||||||||||||.
SARS-CoV         528 SSKRFQPFQQFGRDVSDFTDSVRDPKTSEILDISPCSFGGVSVITPGTNA    577

SARS-CoV-2       590 SNQVAVLYQDVNCTEVPVAIHADQLTPTWRVYSTGSNVFQTRAGCLIGAE    639
                     |::|||||||||||:|..|||||||||.||:||||:|||||:||||||||
SARS-CoV         578 SSEVAVLYQDVNCTDVSTAIHADQLTPAWRIYSTGNNVFQTQAGCLIGAE    627

SARS-CoV-2       640 HVNNSYECDIPIGAGICASYQTQTNSPRRARAHHHHHHHHHH    681
                     ||:.||||||||||||||||.|.:    ..||||||||||||
SARS-CoV         628 HVDTSYECDIPIGAGICASYHTVS----LLRAHHHHHHHHHH    665


#---------------------------------------
#---------------------------------------
```
