# Supplementary material for: Secondary Structures of MERS-CoV, SARS-CoV, and SARS-CoV-2 Spike Proteins Revealed by Infrared Vibrational Spectroscopy
Source: Int J Mol Sci. 2023 May 31;24(11):9550. doi: 10.3390/ijms24119550 (PMC10253540; doi:10.3390/ijms24119550)
Supplement: Supplementary file 1 [file ijms-24-09550-s001.zip › ijms-2282633-supplementary-english.pdf]

International Journal of *Molecular Sciences*

MDPI

Supporting Information

Secondary Structures of MERS-CoV, SARS-CoV, and SARS-CoV-2 Spike Proteins Revealed by Infrared Vibrational Spectroscopy

Annalisa D'Arco [1,2,*], Marta Di Fabrizio [3], Tiziana Mancini [2,*], Rosanna Mosetti [2], Salvatore Macis [2], Giovanna Tranfo [4], Giancarlo Della Ventura [1,5], Augusto Marcelli [1,6], Massimo Petrarca [7,8] and Stefano Lupi [2,7]

1 Laboratori Nazionali Frascati, National Institute for Nuclear Physics (INFN-LNF), Via E. Fermi 54, 00044 Frascati, Italy; giancarlo.dellaventura@uniroma3.it (G.D.V.); augusto.marcelli@lnf.infn.it (A.M.)
2 Department of Physics, University of Rome 'La Sapienza', P.le A. Moro 2, 00185 Rome, Italy; mosetti.1766934@studenti.uniroma1.it (R.M.); salvatore.macis@uniroma1.it (S.M.); stefano.lupi@roma1.infn.it (S.L.)
3 Laboratory of Biological Electron Microscopy, School of Basic Sciences, Institute of Physics, EPFL & Department of Fundamental Microbiology, Faculty of Biology and Medicine, UNIL, 1015 Lausanne, Switzerland; marta.difabrizio@epfl.ch
4 Department of Occupational and Environmental Medicine, Epidemiology and Hygiene, INAIL, Monte Porzio Catone, 00078 Rome, Italy; g.tranfo@inail.it
5 Department of Science, University Rome Tre, Largo San Leonardo Murialdo 1, 00146 Rome, Italy
6 Rome International Centre for Materials Science Superstipes, Via dei Sabelli 119A, 00185 Rome, Italy
7 National Institute for Nuclear Physics Section Rome1, P.le A. Moro 2, 00185 Rome, Italy; massimo.petrarca@uniroma1.it
8 Department of Basic and Applied Sciences for Engineering (SBAI), University of Rome 'La Sapienza', Via Scarpa 16, 00161 Rome, Italy
* Correspondence: annalisa.darco@uniroma1.it (A.D.); tiziana.mancini@uniroma1.it (T.M.)

Citation: D'Arco, A.; Di Fabrizio, M.; Mancini, T.; Mosetti, R.; Macis, S.; Tranfo, G.; Della Ventura, G.; Marcelli, A.; Petrarca, M.; Lupi, S. Secondary Structures of MERS-CoV, SARS-CoV, and SARS-CoV-2 Spike Proteins Revealed by Infrared Vibrational Spectroscopy. *Int. J. Mol. Sci.* **2023**, *24*, 9550. https://doi.org/10.3390/ijms24119550

Academic Editor: Mahmoud Ghomi

Received: 28 February 2023
Revised: 28 April 2023
Accepted: 26 May 2023
Published: 31 May 2023

Copyright: © 2023 by the authors. Licensee MDPI, Basel, Switzerland. This article is an open access article distributed under the terms and conditions of the Creative Commons Attribution (CC BY) license (https://creativecommons.org/licenses/by/4.0/).

S1. Amino acid sequences

S1.1. MERS-CoV S1

YVDVGPDSVKSACIEVDIQQTFFDKTWPRPIDVSKADGIIYPQGRTYSNITITYQGLFPY
QGDHGDMYVYSAGHATGTTPQKLFVANYSQDVKQFANGFVVRIGAAANSTGTVIIS
PSTSATIRKIYPAFMLGSSVGNFSDGKMGRFFNHTLVLLPDGCGTLLRAFYCILEPRSG
NHCPAGNSYTSFATYHTPATDCSDGNYNRNASLNSFKEYFNLRNCTFMYTYNITEDE
ILEWFGITQTAQGVHLFSSRYVDLYGGNMFQFATLPVYDTIKYYSIIPHSIRSIQSDRKA
WAAFYVYKLQPLTFLLDFSVDGYIRRAIDCGFNDLSQLHCSYESFDVESGVYSVSSFEA
KPSGSVVEQAEGVECDFSPLLSGTPPQVYNFKRLVFTNCNYNLTKLLSLFSVNDFTCSQ
ISPAAIASNCYSSLILDYFSYPLSMKSDLSVSSAGPISQFNYKQSFSNPTCLILATVPHNLT
TITKPLKYSYINKCSRLLSDDRTEVPQLVNANQYSPCVSIVPSTVWEDGDYYRKQLSPL
EGGGWLVASGSTVAMTEQLQMGFGITVQYGTDTNSVCPKLEFANDTKIASQLGNCV
EYSLYGVSGRGVFQNCTAVGVRQQRFVYDAYQNLVGYYSDDGNYYCLRACVSVPVS
VIYDKETKTHATLFGSVACEHISSTMSQYSRSTRSMLKRRDSTYGPLQTPVGCVLGLVN
SSLFVEAHHHHHHHHHH

S1.2. SARS-CoV S1

SDLDRCTTFDDVQAPNYTQHTSSMRGVYYPDEIFRSDTLYLTQDLFLPFYSNVTGFHTI
NHTFDNPVIPFKDGIYFAATEKSNVVRGWVFGSTMNNKSQSVIIINNSTNVVIRACNF
ELCDNPFFAVSKPMGTQTHTMIFDNAFNCTFEYISDAFSLDVSEKSGNFKHLREFVFK
NKDGFLYVYKGYQPIDVVRDLPSGFNTLKPIFKLPLGINITNFRAILTAFSPAQDTWGT
SAAAYFVGYLKPTTFMLKYDENGTITDAVDCSQNPLAELKCSVKSFEIDKGIYQTSNFR
VVPSGDVVRFPNITNLCPFGEVFNATKFPSVYAWERKKISNCVADYSVLYNSTFFSTFK

CYGVSATKLNDLCFSNVYADSFVVKGDDVRQIAPGQTGVIADYNYKLPDDFMGCVL
AWNTRNIDATSTGNYNYKYRYLRHGKLRPFERDISNVPFSPDGKPCTPPALNCYWPL
NDYGFYTTTGIGYQPYRVVVLSFELLNAPATVCGPKLSTDLIKNQCVNFNFNGLTGTG
VLTPSSKRFQPFQQFGRDVSDFTDSVRDPKTSEILDISPCSFGGVSVITPGTNASSEVAVL
YQDVNCTDVSTAIHADQLTPAWRIYSTGNNVFQTQAGCLIGAEHVDTSYECDIPIGA
GICASYHTVSLLRAHHHHHHHHHH

S1.3. SARS-CoV-2 S1

VNLTTRTQLPPAYTNSFTRGVYYPDKVFRSSVLHSTQDLFLPFFSNVTWFHAIHVSGTN
GTKRFDNPVLPFNDGVYFASTEKSNIIRGWIFGTTLDSKTQSLLIVNNATNVVIKVCEF
QFCNDPFLGVYYHKNNKSWMESEFRVYSSANNCTFEYVSQPFLMDLEGKQGNFKNL
REFVFKNIDGYFKIYSKHTPINLVRDLPQGFSALEPLVDLPIGINITRFQTLLALHRSYLT
PGDSSSGWTAGAAAYYVGYLQPRTFLLKYNENGTITDAVDCALDPLSETKCTLKSFTV
EKGIYQTSNFRVQPTESIVRFPNITNLCPFGEVFNATRFASVYAWNRKRISNCVADYSV
LYNSASFSTFKCYGVSPTKLNDLCFTNVYADSFVIRGDEVRQIAPGQTGKIADYNYKLP
DDFTGCVIAWNSNNLDSKVGGNYNYLYRLFRKSNLKPFERDISTEIYQAGSTPCNGVE
GFNCYFPLQSYGFQPTNGVGYQPYRVVVLSFELLHAPATVCGPKKSTNLVKNKCVNF
NFNGLTGTGVLTESNKKFLPFQQFGRDIADTTDAVRDPQTLEILDITPCSFGGVSVITP
GTNTSNQVAVLYQDVNCTEVPVAIHADQLTPTWRVYSTGSNVFQTRAGCLIGAEHV
NNSYECDIPIGAGICASYQTQTNSPRRARAHHHHHHHHHH

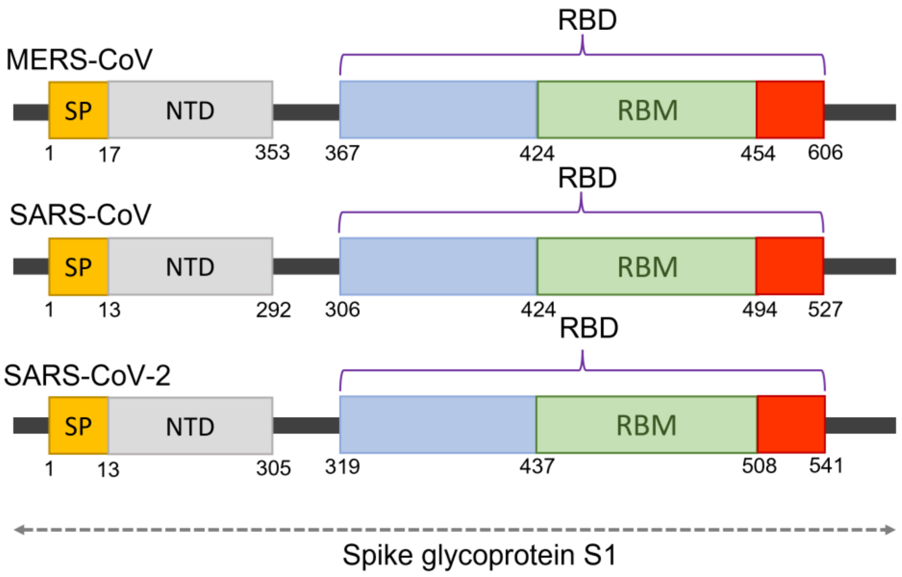

Figure S1. Molecular structures of MERS-CoV, SARS-CoV, and SARS-CoV-2 spike glycoprotein subunits 1.

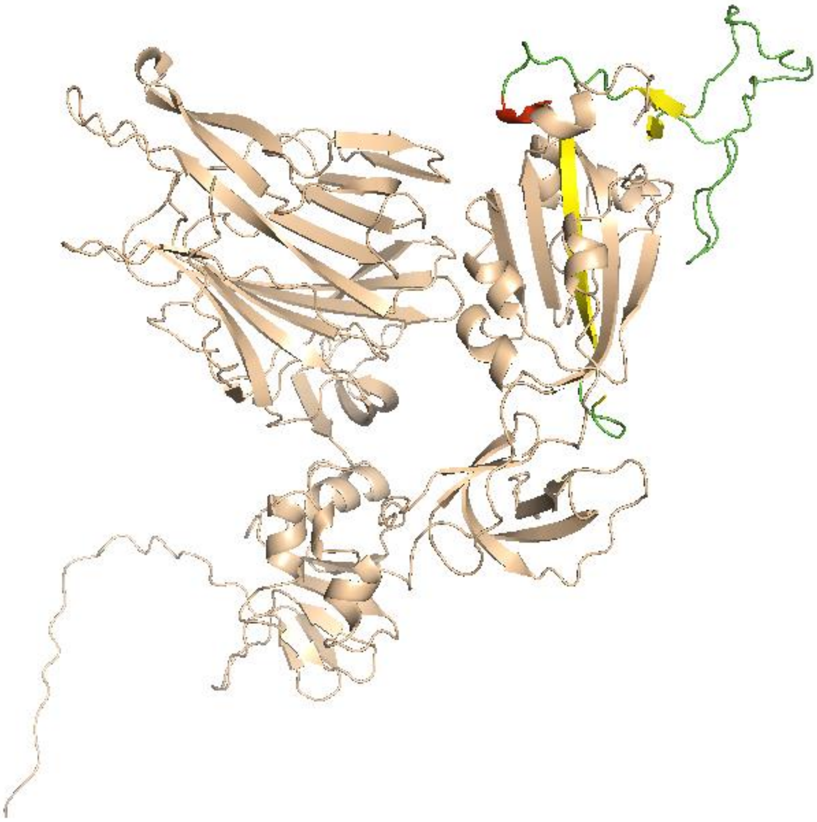

Figure S2. 3D visualization of SARS-CoV-2 S1 fragment in pyMOL. RBM is highlighted with the color palette (red, green, yellow).

S2. Global alignment of S1 proteins

For the protein alignment, we used the Pairwise Sequence Alignment Emboss Needle (https://www.ebi.ac.uk/Tools/psa/emboss_needle/) program. Here, we reported the alignment filenames of the S1, NTD, RBD, and RBM.

- MERS-CoVvsSARS-CoV S1.pdf
- MERS-CoVvsSARS-CoV2 S1.pdf
- SARS-CoVvsSARS-CoV2 S1.pdf
- SARS-CoVvsSARS-CoV2 NTD.pdf
- SARS-CoVvsSARS-CoV2 RBD.pdf
- SARS-CoVvsSARS-CoV2 RBM.pdf

S3. Comparison of SARS-CoV-2 S1 protein at different concentration

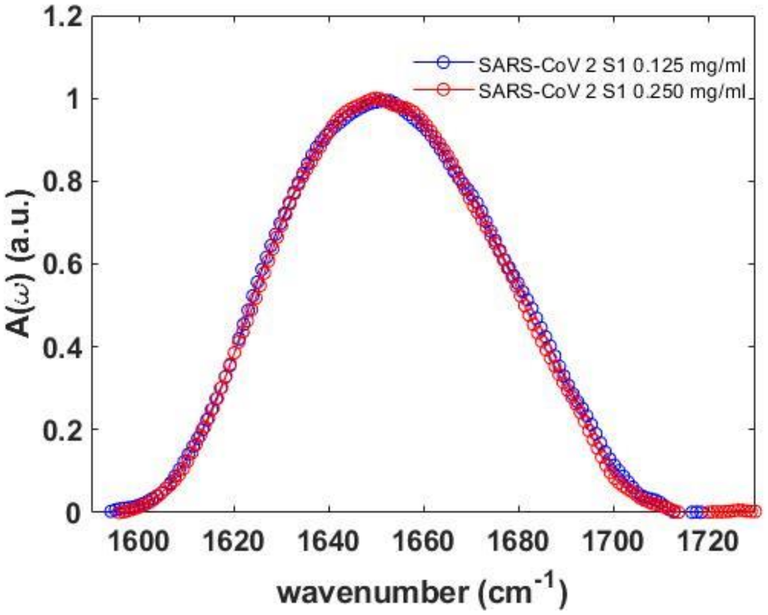

Figure S3. Comparison of normalized amide I SARS-CoV-2 S1 absorption spectra at different concentrations (blue and red circle lines refer to 0.125 mg/ml and 0.250 mg/ml, respectively). An excellent reproducibility of absorption data can be observed.

S4. Secondary structure assignment for SARS-CoV2 S1 protein vs. pH

The main effect of pH concerns, firstly, regards the α-structure (see Table S1), which gave rise to a main absorption band at 1658 cm^{-1} at a pH = 7.4 [1–11]. This component remained nearly constant in frequency (1658–1659 cm^{-1}) for pH when moving from 7.4 to 5.5, while it shifted to a lower frequency (around 1653 cm^{-1}) at alkaline pH levels. Moreover, its intensity remained nearly constant for acid and serological pHs and increased for alkaline pHs (see Figure 4, main text). The band at 1643 cm^{-1} for a 7.4 pH was associated with random coils, which was also influenced by pH variation, and showed blue-shifting to 1645 cm^{-1} for acid and alkaline pHs. Its intensity was maximized at the serological pH value and minimized at the 4.55 pH (see Figure 4, in main text). The extended β-sheet located around 1619 cm^{-1} at the serological and mild acidic and alkaline pHs instead red-shifted for extreme pHs. The other β-sheet components were located between 1620–1640 cm^{-1} and around 1690 cm^{-1} (see Table S1 in supporting information) at a pH=7.4. The band at 1629 cm^{-1} was red-shifted at 1623 cm^{-1} at 4.55 pH levels, suggesting the aggregation of sheets with a large number of strands [1,10], and blue-shifted at 1633 cm^{-1} for alkaline pHs. The absorption of the β-sheet for pH = 4.55 and 5.5 was related to a new band at 1637 cm^{-1}. Finally, the absorption at 1693 cm^{-1} disappeared for extreme alkaline pH values. The total intensity of the β-sheet (see Figure 4), presented a minimum at the serological pH and was maximized at acid pHs. The bands in the 1666–1678 cm^{-1} range, assigned to β-turn structures [1,5,7,8,10,11], were blue-shifted compared to their spectral positions at the 7.4 pH, while a new band appeared around 1686 cm^{-1} for the pH values of 4.55, 8.8, and 11.2. The total intensity of the β-turn structures, which showed a maximum at a pH = 7.4, reduced at both acid and alkaline pHs.

Table S1. Secondary structure assignments for SARS-CoV-2 S1 units as function of pH as derived from IR data analysis. The quantitative structural evaluation (α-helix, β-sheet, β-turn, and random coils) is based on a global fitting through Gaussian components [1–11].

pH 4.55 Peak Frequency [cm⁻¹]	pH 4.55 Relative integrated Intensity [%]	pH 5.5 Peak frequency [cm⁻¹]	pH 5.5 Relative integrated Intensity [%]	pH 7.4 Peak frequency [cm⁻¹]	pH 7.4 Relative integrated Intensity [%]	pH 8.8 Peak frequency [cm⁻¹]	pH 8.8 Relative integrated Intensity [%]	pH 11.2 Peak frequency [cm⁻¹]	pH 11.2 Relative integrated Intensity [%]	Peaks Assignment
1618	15.7	1619	19.2	1619	5.2	1619	11.6	1616	12.4	β-sheet (extended)
1623	8.3	-	-	-	-	-	-	1625	9.1	β-sheet (anti-parallel)
1629	5.1	1629	14.1	1628	11.4	-	-	-	-	β-sheet
-	-	-	-	1633	9.8	1632	23.6	1632	13.1	β-sheet
1637	12.6	1637	9.7	-	-	-	-	1637	6.9	β-sheet
-	-	-	-	1643	12.3	1643	15.1	-	-	Random coil
1646	14.5	1645	15.5	-	-	-	-	1645	14.8	Random coil
1655	18.8	1653	9.7	1650	13.6	1653	12.7	1653	12.2	α-helix
-	-	1659	8.2	1658	15.9	-	-	-	-	α-helix
1663	4.0	-	-	-	-	1663	13.6	1661	10.7	β-turn
-	-	-	-	1666	5.6	-	-	-	-	β-turn
1671	13.3	1667	13.2	-	-	-	-	1669	7.9	β-turn
-	-	-	-	1673	6.7	1674	18.9	1676	7.3	β-turn
-	-	1678	4.9	1678	15.2	-	-	-	-	β-turn
1683	4.4	1684	2.1	-	-	1686	4.3	1686	5.5	β-turn
1690	2.8	1689	2.7	1693	4.2	-	-	-	-	β-sheet (anti-parallel)
1700	0.5	1698	0.7	-	-	-	-	1700	0.2	β-sheet (anti-parallel)

S5. Glycans

The CoV S glycoproteins were densely decorated by heterogeneous N-linked glycans that are used by viral fusion proteins as a shield to counteract the host immune response [12–21]. These oligosaccharides increase the global stability of the proteins [19–21], participating in S folding [22], and work as recognition sites.

Figure S4 shows the absorbance spectra of the S1 glycoproteins of MERS-CoV (A), SARS-CoV (B), and SARS-CoV-2 (C) in the 900–1180 cm^{-1} carbohydrates spectral range. Band overlapping, broadening, and additional bands resulted in quite complex spectra. Despite this, some spectral features were recognized, extrapolated through the second derivative calculation and through a poly-Gaussian fit, and collected in Table S2. In particular, the stretching vibrations of the COH site groups and the glycosidic bonds dominated the region of 900–1180 cm^{-1}. Nevertheless, the few intense IR absorbance peaks at 1035 and 1050 cm^{-1} could be associated with the formation of polymeric chains, as well as the presence of vibrational bands between 900–1000 cm^{-1} and 1150 cm^{-1}, which were attributed to ν(CO) and C-O-C glycosidic linkage, which may serve as markers for the glycosylation degree [23–26].

Other remarkable absorption bands can be observed in Figure S4. Their assignments were based on previous studies in the literature [19–26]. Bands of the skeletal (ring) vibration of glycosidic linkage were observed in the spectral range 900–950 cm^{-1} [23,24]. The peak near 915 cm^{-1} was observed in the spectra of Glc, Fuc, and Man monomers; the 945 cm^{-1} band could be attributed to a Nan monomer contribution. The bands of the ν(CO) mode in COC linkages appeared in the spectral regions encompassing from 965–1000 cm^{-1} and 1130–1160 cm^{-1}. Other strong contributions came from the absorption bands at 994 cm^{-1} and 1035 cm^{-1} [19–21,23–26]. The peak around 990 cm^{-1} was associated with the coupling of in-plane β(CCH), β(CCO) moieties and was observed in the spectra of the Glc, Gal, and Fuc monomers. This was accompanied by bands at 926 cm^{-1} due to the ring and

COH bending modes. The intense peak at 1035 cm^{-1} was a specific spectral feature attributed to the Man and Fuc monomers.

The bands located around 1100 cm^{-1} and 1150 cm^{-1} originated from various couplings of stretching and bending vibrations of the CC and CO bonds. In particular, the absorption near 1150 cm^{-1} was exclusively attributed to the ν(CO) and glycosidic bond. Appreciable differences were not noticeable between the MERS-CoV and SARS-CoV S1 units. In the SARS-CoV-2 S1 unit, slight shifts were observed to a high frequency in the spectral region 1030–1120 cm^{-1}, and a new absorption band appeared at 1015 cm^{-1}. These variations are indicative of different glycan compositions, including the oligosaccharide units and mannose content.

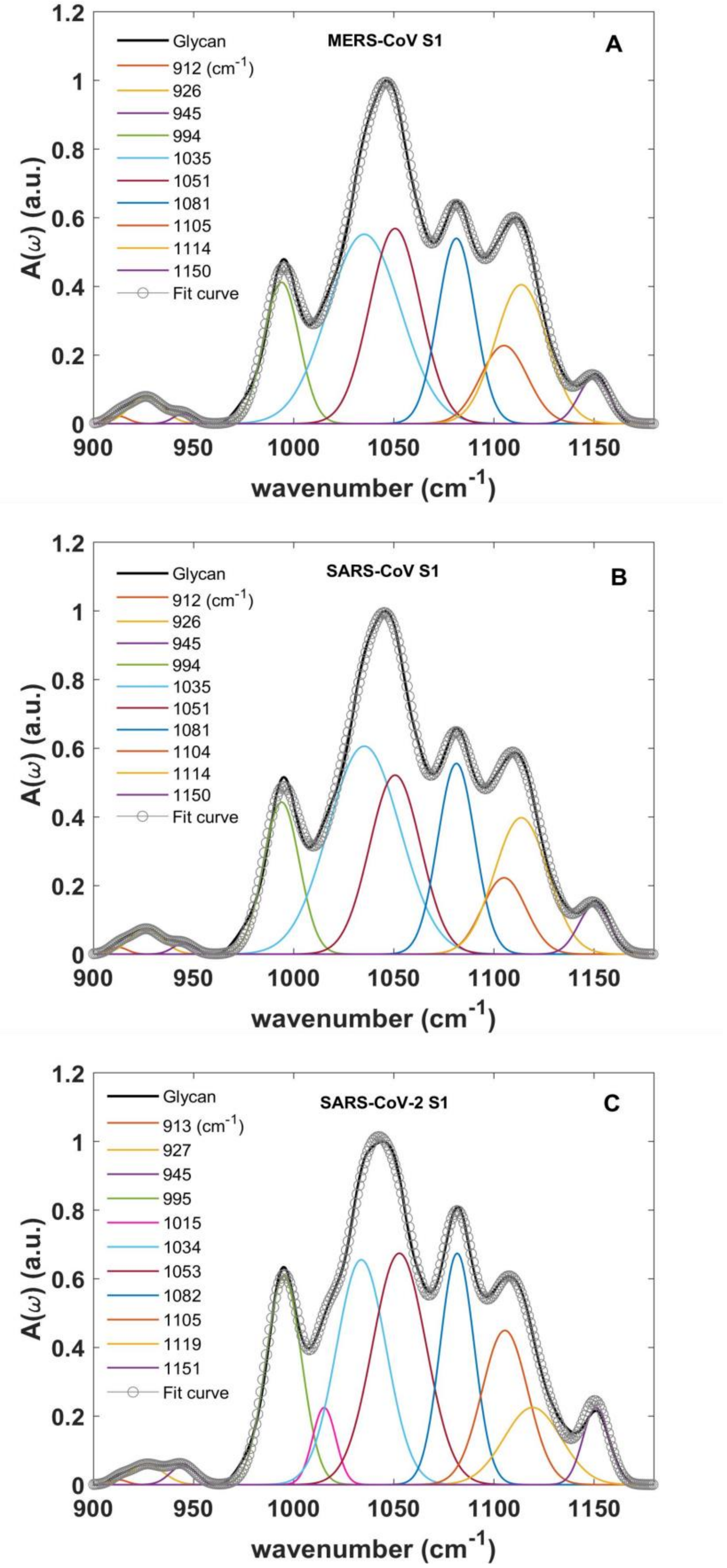

Figure S4. Absorption spectra of glycan band (900–1180 cm^{-1}). Deconvolution of average spectra in the (**A**) MERS-CoV; (**B**) SARS-CoV; and (**C**) SARS-CoV-2 S1 units are reported.

Table S2. Deconvoluted carbohydrates band frequencies and assignments [19–21,23–26].

MERS-CoV (cm^{-1})	SARS-CoV (cm^{-1})	SARS-CoV-2 (cm^{-1})	Assignments
912	913	913	**Glc**, **Fuc, Man** ν(CO), ν(CCH), ν(CO) in glycosidic linkage
926	926	926	ring modes, ν(CO) in glycosidic linkage
945	945	945	**Nan**, ν(CO) in glycosidic linkage
994	994	995	(**Glc, Gal, Fuc**) β(CCH), β(CCO) ν(CO) in glycosidic linkage
-	-	1015	**Man** ν(CC) β(COH)
1035	1035	1033	**Man**,**Fuc** –COH vibration
1051	1051	1052	**Man Gal** ν(CC) ν(CO) β(COH)
1081	1081	1082	**Glc** β(CH)
1103	1104	1107	**Glc**, **Gal** ν(CC) ν(CO) β(COH)
1114	1114	1117	**Man, Fuc**
1150	1151	1150	**Gal, Glc, Nan** ν(CC) ν(CO) in glycosidic linkage

References

1. Barth, A. The infrared absorption of amino acid side chains. *Prog. Biophys. Mol. Biol.* **2000**, *74*, 141–173, https://doi.org/10.1016/s0079-6107(00)00021-3.
2. Piccirilli, F.; Mangialardo, S.; Postorino, P.; Lupi, S.; Perucchi, A. FTIR analysis of the high pressure response of native insulin assemblies. *J. Mol. Struct.* **2013**, *1050*, 159–165, https://doi.org/10.1016/j.molstruc.2013.07.028.
3. Piccirilli, F.; Tardani, F.; D'arco, A.; Birarda, G.; Vaccari, L.; Sennato, S.; Casciardi, S.; Lupi, S. Infrared Nanospectroscopy Reveals DNA Structural Modifications upon Immobilization onto Clay Nanotubes. *Nanomaterials* **2021**, *11*, 1103, https://doi.org/10.3390/nano11051103.
4. Li, H.; Lantz, R.; Du, D. Vibrational Approach to the Dynamics and Structure of Protein Amyloids. *Molecules* **2019**, *24*, 186, https://doi.org/10.3390/molecules24010186.
5. Ricciardi, V.; Portaccio, M.; Perna, G.; Lasalvia, M.; Capozzi, V.; Cammarata, F.P.; Pisciotta, P.; Petringa, G.; Delfino, I.; Manti, L.; et al. FT-IR Transflection Micro-Spectroscopy Study on Normal Human Breast Cells after Exposure to a Proton Beam. *Appl. Sci.* **2021**, *11*, 540, https://doi.org/10.3390/app11020540.
6. Surewicz, W.K.; Leddy, J.J.; Mantsch, H.H. Structure, stability, and receptor interaction of cholera toxin as studied by Fourier-transform infrared spectroscopy. *Biochemistry* **1990**, *29*, 8106–8111, https://doi.org/10.1021/bi00487a017.
7. Jackson, M.; Mantsch, H.H. Beware of proteins in DMSO. *Biochim. et Biophys. Acta (BBA) - Protein Struct. Mol. Enzym.* **1991**, *1078*, 231–235, https://doi.org/10.1016/0167-4838(91)90563-f.
8. Yang, H.; Yang, S.; Kong, J.; Dong, A.; Yu, S. Obtaining information about protein secondary structures in aqueous solution using Fourier transform IR spectroscopy. *Nat. Protoc.* **2015**, *10*, 382–396, https://doi.org/10.1038/nprot.2015.024.
9. Pelton, J.T.; McLean, L.R. Spectroscopic Methods for Analysis of Protein Secondary Structure. *Anal. Biochem.* **2000**, *277*, 167–176, https://doi.org/10.1006/abio.1999.4320.
10. Barth, A. Infrared spectroscopy of proteins. *Biochim. Biophys. Acta Bioenerg.* **2007**, *1767*, 1073–1101, https://doi.org/10.1016/j.bbabio.2007.06.004.
11. Seshadri, S.; Khurana, R.; Fink, A.L. Fourier transform infrared spectroscopy in analysis of protein deposits. **1999**, *309*, 559–576, https://doi.org/10.1016/s0076-6879(99)09038-2.
12. Walls, A.C.; Tortorici, M.A.; Frenz, B.; Snijder, J.; Li, W.; A Rey, F.; DiMaio, F.; Bosch, B.-J.; Veesler, D. Glycan shield and epitope masking of a coronavirus spike protein observed by cryo-electron microscopy. *Nat. Struct. Mol. Biol.* **2016**, *23*, 899–905, https://doi.org/10.1038/nsmb.3293.
13. Walls, A.; Tortorici, M.A.; Bosch, B.-J.; Frenz, B.; Rottier, P.J.M.; DiMaio, F.; Rey, F.A.; Veesler, D. Crucial steps in the structure determination of a coronavirus spike glycoprotein using cryo-electron microscopy. *Protein Sci.* **2016**, *26*, 113–121, https://doi.org/10.1002/pro.3048.
14. Walls, A.C.; Tortorici, M.A.; Snijder, J.; Xiong, X.; Bosch, B.-J.; Rey, F.A.; Veesler, D. Tectonic conformational changes of a coronavirus spike glycoprotein promote membrane fusion. *Proc. Natl. Acad. Sci. USA* **2017**, *114*, 11157–11162, https://doi.org/10.1073/pnas.1708727114.

15. Xiong, X.; Tortorici, M.A.; Snijder, J.; Yoshioka, C.; Walls, A.C.; Li, W.; McGuire, A.T.; Rey, F.A.; Bosch, B.-J.; Veesler, D. Glycan Shield and Fusion Activation of a Deltacoronavirus Spike Glycoprotein Fine-Tuned for Enteric Infections. *J. Virol.* **2018**, *92*, https://doi.org/10.1128/jvi.01628-17.
16. Andersen, K.G.; Rambaut, A.; Lipkin, W.I.; Holmes, E.C.; Garry, R.F. The proximal origin of SARS-CoV-2. *Nat. Med.* **2020**, *26*, 450–452, https://doi.org/10.1038/s41591-020-0820-9.
17. Shajahan, A.; Supekar, N.T.; Gleinich, A.S.; Azadi, P. Deducing the N- and O-glycosylation profile of the spike protein of novel coronavirus SARS-CoV-2. *Glycobiology* **2020**, *30*, 981–988, https://doi.org/10.1093/glycob/cwaa042.
18. Imperiali, B.; E O'connor, S. Effect of N-linked glycosylation on glycopeptide and glycoprotein structure. *Curr. Opin. Chem. Biol.* **1999**, *3*, 643–649, https://doi.org/10.1016/s1367-5931(99)00021-6.
19. Wormald, M.R.; A Dwek, R. Glycoproteins: glycan presentation and protein-fold stability. *Structure* **1999**, *7*, R155–R160, https://doi.org/10.1016/s0969-2126(99)80095-1.
20. Cui, J.; Li, F.; Shi, Z.-L. Origin and evolution of pathogenic coronaviruses. *Nat. Rev. Microbiol.* **2019**, *17*, 181–192, https://doi.org/10.1038/s41579-018-0118-9.
21. Petersen, E.; Koopmans, M.; Go, U.; Hamer, D.H.; Petrosillo, N.; Castelli, F.; Storgaard, M.; Al Khalili, S.; Simonsen, L. Comparing SARS-CoV-2 with SARS-CoV and influenza pandemics. *Lancet Infect. Dis.* **2020**, *20*, e238–e244, https://doi.org/10.1016/s1473-3099(20)30484-9.
22. Pallesen, J.; Wang, N.; Corbett, K.S.; Wrapp, D.; Kirchdoerfer, R.N.; Turner, H.L.; Cottrell, C.A.; Becker, M.M.; Wang, L.; Shi, W.; et al. Immunogenicity and structures of a rationally designed prefusion MERS-CoV spike antigen. *Proc. Natl. Acad. Sci. USA* **2017**, *114*, E7348–E7357, https://doi.org/10.1073/pnas.1707304114.
23. Kačuráková, M.; Mathlouthi, M. FTIR and laser-Raman spectra of oligosaccharides in water: characterization of the glycosidic bond. *Carbohydr. Res.* **1996**, *284*, 145–157, https://doi.org/10.1016/0008-6215(95)00412-2.
24. Wiercigroch, E.; Szafraniec, E.; Czamara, K.; Pacia, M.Z.; Majzner, K.; Kochan, K.; Kaczor, A.; Baranska, M.; Malek, K. Raman and infrared spectroscopy of carbohydrates: A review. *Spectrochim. Acta Part A Mol. Biomol. Spectrosc.* **2017**, *185*, 317–335, https://doi.org/10.1016/j.saa.2017.05.045.
25. Khajehpour, M.; Dashnau, J.L.; Vanderkooi, J.M. Infrared spectroscopy used to evaluate glycosylation of proteins. *Anal. Biochem.* **2006**, *348*, 40–48, https://doi.org/10.1016/j.ab.2005.10.009.
26. Lisowska, E. The role of glycosylation in protein antigenic properties. *Cell. Mol. Life Sci.* **2002**, *59*, 445–455, https://doi.org/10.1007/s00018-002-8437-3.

Disclaimer/Publisher's Note: The statements, opinions and data contained in all publications are solely those of the individual author(s) and contributor(s) and not of MDPI and/or the editor(s). MDPI and/or the editor(s) disclaim responsibility for any injury to people or property resulting from any ideas, methods, instructions or products referred to in the content.
